# Significant Differences in Personality Styles of Securely and Insecurely Attached Psychotherapists: Data, Reflections and Implications

**DOI:** 10.3389/fpsyg.2020.00611

**Published:** 2020-04-21

**Authors:** Burkhard Peter, Eva Böbel

**Affiliations:** Department of Psychology, Ludwig-Maximilians Universität München, Munich, Germany

**Keywords:** psychotherapist, personality, attachment, PSDI, RSQ, therapist variable

## Abstract

This is a contribution to the research on the therapist variable aiming to improve effectiveness of psychotherapy. It is shown that attachment styles shape personality styles of psychotherapists in a favorable or unfavorable manner. Data on personality (PSDI) and attachment (RSQ) styles was collected from 430 psychological psychotherapists of the DACH countries using an online survey. The 88 insecurely attached psychotherapists differed significantly from their 342 securely attached colleagues in 9 of 14 personality styles: They were – even though well within normal range – more paranoid, borderline, schizoid, dependent, negativistic, self-sacrificing, avoidant, and depressive, as well as less optimistic. This corresponds to results of other researchers. Data regarding their effectiveness was not available. It is argued that a secure attachment style predispose to be a good psychotherapist. Yet, insecurely attached psychotherapists possibly compensate their adverse traits through self-therapy, continuous education, and supervision.

## Introduction

Treatment outcome in psychotherapy is mostly, but not exclusively, associated with factors related to the patient (e.g., severity and chronicity of the disorder), but also with therapist variables, for example, the therapist’s personality and interpersonal skills (Beutler et al., 2004; Lambert, 2013). In an extensive meta-analysis, therapist effects accounted for about 5% of the variance in treatment outcome which seems small only on first sight (Baldwin and Imel, 2013). Looking closer, the therapist’s influence is more profound, as he or she seems to be the crucial factor in one of the most researched and robust single predictors of treatment success, which is the therapeutic alliance (Baldwin et al., 2007; Del Re et al., 2012). The term therapeutic or working alliance refers to the collaborative partnership between client and therapist in reaching the treatment goals (Bordin, 1979; Horvath and Greenberg, 1994). It was found to account for 8% of the variance in treatment outcome in a recent meta-analysis (Flückiger et al., 2018), and therapist variability appears to be the crucial factor in alliance quality as opposed to patient variability (Del Re et al., 2012). However, it is not quite clear how therapists actually foster a good working alliance. A systematic review of therapists’ influence on treatment outcome in psychodynamic therapies found that therapists’ interpersonal functioning showed the strongest evidence for directly affecting outcome (Lingiardi et al., 2017). Likewise, Heinonen and Nissen-Lie (2019) concluded in their most recent review that more effective psychotherapists seem to be characterized by interpersonal capacities which are rooted in their personal lives and attachment history.

### Attachment in Therapists

Interpersonal patterns as well as therapist attachment styles and personal caregiver history appear to be related to therapeutic alliance, thus indirectly influencing treatment outcome. Attachment styles (orientations or patterns) are assumed to be formed and fed by childhood experiences with caregivers, represented in so-called “internal working models” according to Bowlby’s attachment theory (Bowlby, 1969). In adults, they are rated in interviews, e.g., by the Adult Attachment Interview, AAI (George et al., 1985), or by self-rating scales such as the Relationship Scales Questionnaire, RSQ (Griffin and Bartholomew, 1994b). While there is extensive literature on client attachment styles and their impact on the course and outcome of treatment (for an overview see Mallinckrodt and Jeong, 2015; Levy et al., 2018), the literature on therapist attachment styles is sparse. This is unsurprising because when focusing directly on the association of therapist attachment and therapeutic alliance quality in treatment, results are inconclusive, probably also due to methodological issues and the complexity of this relationship (Degnan et al., 2016; Steel et al., 2018). In a systematic review of 11 eligible treatment studies, Degnan et al. (2016) found some evidence that therapists’ secure attachment was related to stronger alliances with their clients (and also to a better outcome), but also found evidence for significant interactions between therapist and client attachment patterns. Accordingly, Steel et al. (2018), in 19 papers, revealed that therapist attachment affects therapeutic relationship quality (as observed in client-rated evaluation), therapist negative countertransference, empathy, and problems in therapy. Interaction effects between client and therapist attachment style were corroborated. However, the relationship between therapist attachment style and therapeutic alliance is not straightforward, as there is also some evidence that therapists and clients with oppositional attachment styles reported more favorable alliances. Petrowski et al. (2011), e.g., reported that anxiously attached clients viewed the relationship with a more avoidant therapist as more helpful. Fuertes et al. (2019) found that therapists with higher attachment anxiety and avoidance reported more difficulties in seeing their clients in ways that benefited them, but this was not associated with the perception of the clients regarding the relationship and therapy progress. Generally, it is likely that the complexity of clients’ presenting problems, coupled with the interaction between client-therapist attachment styles, influences the therapeutic alliance (Mikulincer et al., 2013; Bucci et al., 2015). Schauenburg et al. (2010), for example, reported higher attachment security of psychotherapists being associated with better alliance and outcome only in severely impaired patients. Taken together, because of their relevance for treatment outcome, therapist variables generally deserve more attention, with therapists’ interpersonal functioning and attachment styles having been identified as especially noteworthy (Heinonen and Nissen-Lie, 2019).

Few studies compared therapists’ attachment styles directly to population norms. 56–58% of non-clinical adults in Western samples were found to be securely attached (Bakermans-Kranenburg and van IJzendoorn, 2009). In therapists, the rate of secure attachment patterns seems roughly equal to this proportion: In the respective studies, about 50–70% of therapists were classified as securely attached (Dinger, 2016). Schauenburg et al. (2006) reported 45.2%, Schauenburg et al. (2010) 61.3%, and Dinger et al. (2009) about 50%, Talia et al. (2018) 64% of securely attached psychodynamic psychotherapists, as rated using the AAI. However, some studies imply that the proportion of securely attached therapists may be higher: In a sample of 50 German trainees, for example, 78% were rated to be securely attached (Taubner et al., 2014b) and 72% of 290 licensed North American psychologists (Fleischman and Shorey, 2016).

### Personality in Therapists

Therapists’ personality traits in particular have scarcely been researched so far (Lingiardi et al., 2017) but are also likely to be of influence in building a good working alliance (Chapman et al., 2009; Taber et al., 2011) as well as other therapeutic skills. Although there is a substantial amount of literature on which qualities and skills therapists should present (Keenan and Rubin, 2016), there is surprisingly little research on what therapists are actually like. Regarding personality traits, most research so far has focused on how personality traits are associated with choosing a particular therapeutic approach or identifying with a particular theoretical framework (Arthur, 2001, for a review of early research). For example, some studies reported that therapists/trainees with a psychodynamic orientation presented more “Openness to Experience” (measured with the NEO Five Factor Inventory or its revised version; Costa and McCrae, 1992) compared to those with a behavioral-cognitive orientation (Poznanski and McLennan, 2003; Buckman and Barker, 2010; Taubner et al., 2014a). However, only few studies inform on therapists’ personality profiles in relation to normative scores from the general population. One exception is Boswell et al. (2009) who found openness and neuroticism scores in 46 United States-American graduate student therapists in-training to be in the high range, while extraversion, agreeableness, and conscientiousness were in the average range.

**FIGURE 1:**
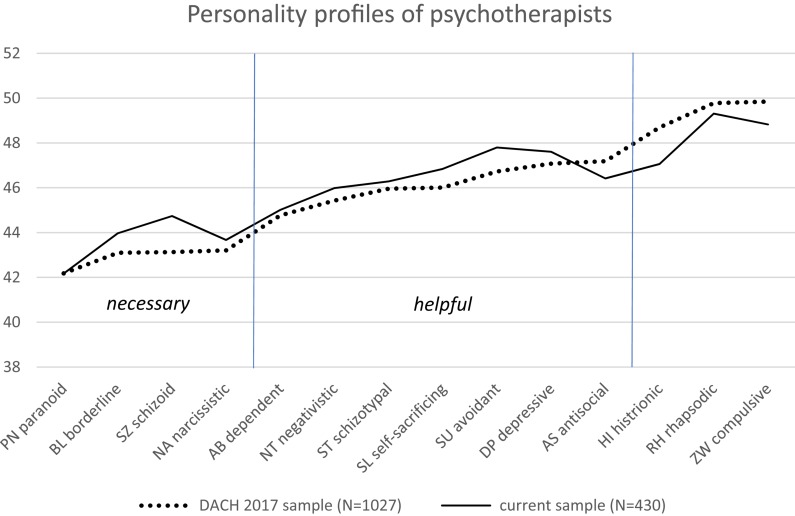
Personality profile of all (*N* = 1,027) psychotherapists of Germany (D), Austria (A), and Switzerland (CH) (DACH countries) surveyed in 2015 [dotted line, adapted from Peter et al. (2017)] in comparison to the current sample (*N* = 430, solid line) and the normative sample (*N* = 3,392). The average scores of the DACH psychotherapists and the current sample were in the normal range of 40 and 60, but consistently below the mean average of *T*-value 50. Peter et al. (2017) interpreted the low values of the personality styles PN, BL, SZ, NA as *necessary* for forming a good therapeutic alliance, and AB until AS as *helpful* in the course of psychotherapy.

In 2015, to address this question, we aimed to achieve a representative survey of therapists from the three German-speaking countries Germany (D), Austria (A), and Switzerland (CH) (DACH-countries) (Peter et al., 2017). In total, 1,027 psychotherapists (average age 54 years; 71% female) anonymously completed the short version of the Personality Style and Disorder Inventory (PSDI; Kuhl and Kazén, 2009) which assesses personality styles partly based on non-pathological equivalents of classifiable personality disorders (see Table 1). Results showed that although their average personality profile was within the normal range of 40–60 T-scores, therapists scored significantly below the normative mean of 50 in 11 of the 14 personality styles (see Figure 1). Therapists showed especially low levels of willful/paranoid (PN), spontaneous/borderline (BL), reserved/schizoid (SZ), and ambitious/narcissistic (NA) styles, with large effect sizes. So, either therapists painted themselves in a quite positive light, or – and this is how we interpret the results – this sample of experienced clinicians (with nearly 20 years of professional practice on average) was basically free from pathological personality styles and demonstrated a personality profile which is – in our interpretation – *necessary* for building a good therapeutic relationship. They were able to put their personal opinions aside, show empathy and appreciation, open themselves to the emotional experience of the patient, and provide a trusting relationship. Medium effect sizes, i.e., moderate differences below the normative mean, were found in the following personality styles: Loyal/dependent (AB), critical/negativistic (NA), intuitive/schizotypal (ST), unselfish/self-sacrificing (SL), self-critical/avoidant (SU), passive/depressive (DE), and assertive/antisocial (AS). We interpreted these styles as equally indicative of the professional social skills of psychotherapists, i.e., they were neither submissive nor critical, neither excessively helpful nor too self-critical, not passive, but also not too self-assertive.

**TABLE 1:** The 14 scales of the personality styles and disorders inventory (PSDI; Kuhl and Kazén, 2009).

**PSDI-scale ^a^**	**Example**
PN willful/**paranoid**	“Most people mean well” (negatively coded)
BL spontaneous/**borderline**	“My feelings often change abruptly and impulsively”
SZ reserved/**schizoid**	“I always keep my distance to other people”
NA ambitious/**narcissistic**	“The idea of being a famous personality appeals to me”
AB loyal/**dependent**	“I need a lot of love and acceptance”
NT critical/negativistic	“I have frequently been persecuted by bad luck”
ST intuitive/**schizotypal**	“There are supernatural force”
SL unselfish/self-sacrificing	“I am more concerned with other people’s worries than my own needs”
SU self-critical/**avoidant**	“Criticism hurts me quicker than it does to others”
DP passive/depressive	“I often feel low and feeble”
AS assertive/**antisocial**	“If people turn against me I can get them down”
HI charming/**histrionic**	“My good moods are very contagious to others”
RH optimistic/rhapsodic	“I am an invincible optimist”
ZW conscientious/**compulsive**	“Consistency and firm principles define my life”

*^*a*^ DSM-5 or ICD-10 equivalents are in bold print.*

### Personality and Attachment – Combined Measures

Only few studies measured both, personality and attachment. Petronzi and Masciale (2015), using the HEXACO-60 (Ashton and Lee, 2009), and the Relationships Questionnaire (RQ; Bartholomew and Horowitz, 1991), examined whether people’s personality and attachment styles would predict their preferences for one of three psychotherapeutic approaches. In an online sample (*N* = 209), they found that openness and secure attachment predicted a preference for psychodynamic psychotherapy, while fearful attachment predicted a preference for cognitive-behavioral psychotherapy, which is in contrast to the online survey of Fleischman and Shorey (2016) who found that psychodynamic therapists reported higher levels of attachment anxiety than cognitive-behavioral therapists.

Sherry et al. (2007), in a sample of 273 undergraduates, correlated the RSQ with 10 personality scales on the Millon Clinical Multiaxial Inventory-III (Millon et al., 1997). RSQ was able to predict seven personality styles. Insecure attachment was related to paranoid, borderline, schizoid, dependent, schizotypal, and avoidant personality styles, while secure attachment was related to the histrionic personality style. No relationships were found regarding the narcissistic, antisocial and compulsive personality styles.

In a sample of (also) 273 undergraduates (73% female), Both and Best (2017) used the combined measures of RQ and RSQ and the NEO-PI-R (Costa and McCrae, 1992). On both measures, higher scores on secure attachment were associated with positive personality characteristics such as low neuroticism and being outgoing, while insecure patterns were associated with the opposite as well as personality facets of low trust and high depression (see Table 3). The personality factors and facets of the three insecure attachment styles can be seen in Table 3, which simultaneously gives an overview of the four-category model of attachment styles according to Bartholomew and Horowitz (1991). In summary, people with higher anxiety show higher neuroticism and lower agreeableness, and people with higher avoidance show lower levels of trust.

Finally, it should be noted that Schauenburg et al. (2006) reported considerably higher depression scores (using the BDI; Hautzinger et al., 1995) in their 45.2% insecurely attached psychodynamically oriented psychotherapists in contrast to the securely attached.

### Purpose of the Current Study

Having found meaningful personality profiles in psychotherapists in 2015 (Peter et al., 2017), we were now, in 2017/18, interested in whether there would also be meaningful differences in the attachment styles of these DACH psychotherapists. With the aim of replicating and expanding our previous findings, we again addressed this original DACH-sample and assessed both attachment and personality styles. In order to increase the number of participants, we also addressed another pool of professionals of whom we had the email addresses at hand. For both samples, (1) we expected differences in most if not all personality styles between the securely and insecurely attached psychotherapists. (2) We expected that our data would be similar to those reported by Sherry et al. (2007); Petronzi and Masciale (2015), and Both and Best (2017). And (3) we were simply curious as to whether our sample would contain more securely or insecurely attached psychotherapists, as the above referred studies differed considerably in this regard.

## Materials and Methods

### Sample and Recruitment Procedures

In late 2017/early 2018, we once again contacted those approx. 4,600 previously addressed psychotherapists from the DACH countries via e-mail. Back in 2015, *N* = 1,027 had answered, but this time only *N* = 267, *n* = 189 female, and *n* = 78 male, with an average age of 57.1 (SD = 9.39) years (hereafter referred to as DACH 2) responded. The reasons so few individuals replied may be as follows:

• This time we asked them to fill out the Personality Styles and Disorders Inventory (PSDI), as well as the RSQ, so the processing time doubled;
• about 200 email addresses were no longer valid;
• some respondents had already responded in 2015. (Unfortunately, we do not know how many of those from 2015 responded once more or how many responded the first time. So this is not a proper replication).

At the same time, we also emailed the same questionnaires (PSDI and RSQ) to about 3,500 additional professionals via the MEG hypnosis-3list-server (hereafter referred to as MEG 2). Of these, we obtained *N* = 500 evaluable data from *n* = 371 women and *n* = 129 men with an average age of 52.2 (SD = 10.2) years. The clinical practitioners in question were either psychological (*n* = 212) or medical (*n* = 47) psychotherapists, or were other clinical practitioners using hypnosis (*n* = 241; including medical doctors without psychotherapy training, social pedagogues, etc.). For parallelization purposes, we used the data of the psychological psychotherapists only; these were *n* = 218 for the DACH 2 and *n* = 212 for the MEG 2 sample. As there were some recruitment differences for the two samples with regard to the context of hypnosis which are addressed in another publication by Peter and Böbel (2020), we firstly checked whether to treat both samples separately. As the comparison of the two samples’ data revealed no significant differences (see Table 2) we combined them to one sample of *N* = 430.

**TABLE 2:** Descriptive data and results of *t*-tests (by equal variances) of the two sub-samples MEG 2 and DACH 2.

**Personality styles**	**Sub-sample**	***n***	***Mean of T-values***	***SD***	***SE of mean***	***T***	***df***	p* (two-tailed)	*Mean difference* ($\bar{x}{}_{1}$−$\bar{x}{}_{2}$)**	SE of difference ($\bar{x}{}_{1}$−$\bar{x}{}_{2}$)**
PN willful/**paranoid**	MEG 2	212	41.41	8.37	0.58	–1.84	428	0.067	–1.49	0.815
	DACH 2	218	42.90	8.51	0.58					
BL spontaneous/**borderline**	MEG 2	212	44.32	5.74	0.39	1.29	428	0.197	0.68	0.530
	DACH 2	218	43.63	5.24	0.35					
SZ reserved/**schizoid**	MEG 2	212	44.54	9.53	0.65	–0.43	428	0.669	–0.39	0.916
	DACH 2	218	44.93	9.47	0.64					
NA ambitious/**narcissistic**	MEG 2	212	44.27	7.59	0.52	1.63	428	0.104	1.19	0.733
	DACH 2	218	43.08	7.61	0.52					
AB loyal/**dependent**	MEG 2	212	45.15	7.56	0.52	0.34	428	0.732	0.27	0.775
	DACH 2	218	44.89	8.47	0.57					
NT critical/negativistic	MEG 2	212	46.42	7.48	0.51	1.25	428	0.214	0.86	0.692
	DACH 2	218	45.56	6.86	0.46					
ST intuitive/**schizotypal**	MEG 2	212	46.93	7.46	0.51	1.71	428	0.087	1.27	0.740
	DACH 2	218	45.66	7.88	0.53					
SL unselfish/self-sacrificing	MEG 2	212	46.85	8.16	0.56	0.02	428	0.988	0.01	0.804
	DACH 2	218	46.84	8.51	0.58					
SU self-critical/**avoidant**	MEG 2	212	48.73	7.60	0.52	2.49	428	0.013	1.85	0.741
	DACH 2	218	46.89	7.76	0.53					
DP passive/depressive	MEG 2	212	47.71	6.93	0.48	0.33	428	0.745	0.22	0.679
	DACH 2	218	47.49	7.14	0.48					
AS assertive/**antisocial**	MEG 2	212	46.04	7.64	0.52	–0.98	428	0.326	–0.75	0.762
	DACH 2	218	46.78	8.14	0.55					
HI charming/**histrionic**	MEG 2	212	47.79	8.94	0.61	1.69	428	0.092	1.44	0.855
	DACH 2	218	46.35	8.80	0.60					
RH optimistic/rhapsodic	MEG 2	212	49.96	8.19	0.56	1.59	428	0.113	1.29	0.807
	DACH 2	218	48.68	8.54	0.58					
ZW conscientious/**compulsive**	MEG 2	212	48.56	7.72	0.53	–0.69	428	0.488	–0.54	0.780
	DACH 2	218	49.09	8.42	0.57					

* significant if *p* < 0.0018. ** $\bar{x}{}_{1}$ = MEG 2, $\bar{x}{}_{2}$ = DACH 2. DSM-5 or ICD-10 equivalents are in bold print.

### Instruments

#### RSQ for Attachment Styles

The German translation of the Relationship Scale Questionnaire (RSQ) was used. The RSQ is a self-rating instrument developed by Griffin and Bartholomew (1994b) which is based on attachment theory (Bowlby, 1969) and yields two underlying attachment dimensions: view of self and of other (positive and negative), and the dimensions of “anxiety (of separation)” and “avoidance (of closeness)” (see Table 3). The RSQ consists of 30 items which are rated on a five-point Likert scale. The items ask about one’s feelings in “close relationships” and attitudes concerning aspects of those relationships, such as closeness, dependency, or the feeling of being loved, somewhat explicitly as if regarding a “romantic partner.” Griffin and Bartholomew (1994a) reported suitable convergent and discriminate validity of these two dimensions, and moderate to high test-retest reliability (between 0.81 and 0.84 for view of self and between 0.72 and 0.85 for view of other). Scharfe and Bartholomew (1994) reported test-retest reliabilities between 0.39 and 0.58. Griffin and Bartholomew (1994b) reported internal consistencies ranging from 0.31 to 0.47. In a sample of Brussoni et al. (2000), internal consistencies of the RSQ scores lay at Cronbach’s α = 0.51, 0.48, 0.70, and 0.76 (for the secure, preoccupied, dismissing, and fearful styles, respectively). The German translation was provided and published by Mestel (1994). An analysis of this German version by means of a confirmatory factor analysis Steffanowski et al. (2001) suggested four factors: anxiety (of separation), avoidance (of closeness), lack of trust and (desire for) independence. All four scales showed good internal consistency within a German speaking sample in Steffanowski et al.’s (2001) study using Cronbach’s alpha (−0.81 for anxiety, 0.77 for avoidance and lack of trust, and −0.72 for independence). Steffanowski et al. (2001) used the values of a non-clinical sample to determine appropriate cut-off values for clinical significance: 2.88 points beyond the mean of the anxiety scale and 2.75 points above the mean of the avoidance scale. Low values on both scales indicate secure attachment; high anxiety and low avoidance indicate an insecure-preoccupied attachment style; low anxiety and high avoidance are presumed to present an insecure-dismissing attachment style. Finally, high scores on both scales (that is, anxiety and avoidance in equal measure) indicate the insecure-fearful attachment style (Table 3). To achieve enough power, we pooled the three insecure attachment styles, so in the following, we compare the two styles of secure and insecure attachment.

**TABLE 3:** Four-category model of attachment styles according to Bartholomew and Horowitz (1991), the two views (positive and negative) of self and other and the dimensions of “anxiety (of separation)” and “avoidance (of closeness).”

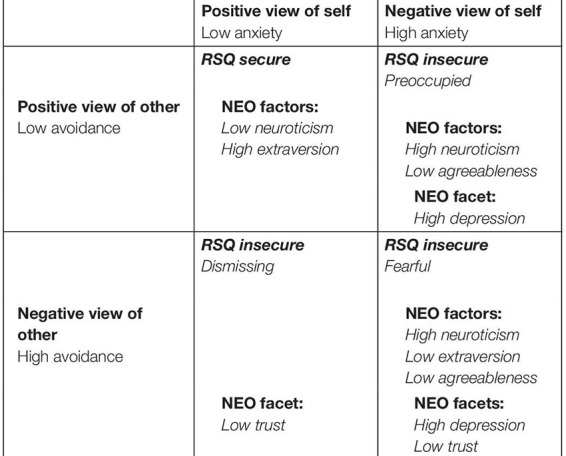

*Included: the NEO-PI-R Factors and Facets of Both and Best (2017). DSM-5 or ICD-10 equivalents are in bold print.*

#### PSDI for Personality Styles

The original German version of the Personality Styles and Disorders Inventory (PSDI; Kuhl and Kazén, 2009) was used in its shortened version (PSDI-S) with 56 items. The PSDI is a self-assessment tool that captures the relative markedness of 14 personality styles, including 10 styles that can be considered non-pathological equivalents of the personality disorders described in DSM-IV and ICD-10 (see Table 1). The answer scales are four-level with the poles being “does not apply at all” and “fully applies.” The raw values are converted into standardized *T*-values with 50 being the mean value. The normalization sample of the PSDI-S consisted of 3,392 participants (1,763 women and 1,629 men) between the ages 12–82, who had different occupations (students, managers, regular employees, and homemakers). The PSDI-S is standardized, provides objective procedures and analyses, and mostly has satisfactory reliability (Cronbach’s α = 0.69–0.84 which is slightly below Cronbach’s α = 0.75–0.85 of the long version of the PSDI; unpublished data provided by Kazén, unpublished). The validity of the PSDI long version has been demonstrated in several studies, e.g., medium to high correlations with other inventories such as the NEO-FFI. (We refer to this issue in Table 6 and in the “Discussion” section.) According to many studies including ours (Peter and Böbel, 2020; Peter et al., 2014, Peter et al., 2017), the PSDI is a very well-suited instrument to research personality.

Please note: In order to illustrate the different personality styles, we include the names and descriptions of the respective personality disorders. For example, instead of merely referring to a personality style as “willful,” we refer to it as “willful/paranoid (PN).” This is only for illustrative purposes as these additional nomenclatures are the commonly used terms in the clinical field. The mean scores of our sample, however, are well within the range established by Kuhl and Kazeìn (2009) as normal expressions of personality styles. As abbreviations of the styles, in this paper we use the German characters, as we did so in all other publications: The German word for “dependent” for example is “abhängig”; therefore, the abbreviation for loyal/dependent is “AB.”

### Data Analyses

The data collected using SoSciSurvey were loaded directly into SPSS (version 23). The confidence intervals for effect sizes were obtained with the statistical software R (Version 3.2.2). Hypotheses were tested using *t*-tests. None of the PSDI-S scales were normally distributed. However, as *t*-test is considered to be robust against violations of the assumption of normality, we chose to refrain from using non-parametric tests for two reasons: Firstly, for having more power to detect existing differences, and secondly, for having the possibility to compute confidence intervals that allow us to gauge the magnitude of these differences. Levene tests were used to assess homogeneity of variances. Because of multiple comparisons, the threshold for significance was set at *p* = 0.0018 after Bonferroni correction.

## Results

**FIGURE 2:**
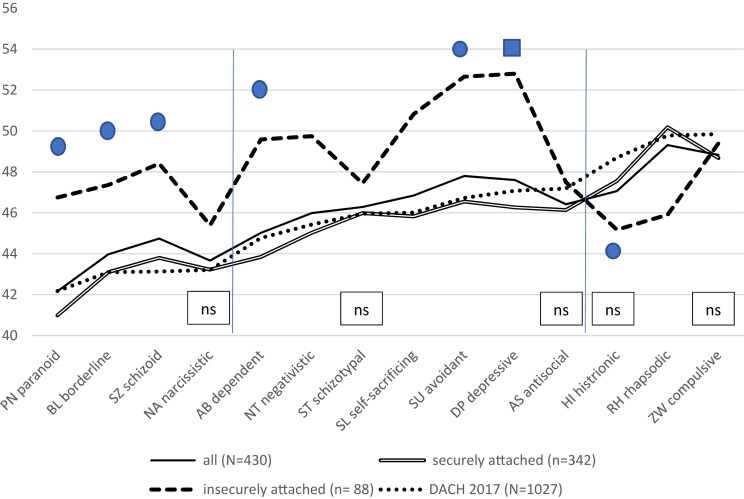
All securely (*n* = 342; drawn through double line) and insecurely (*n* = 88; bold interrupted line) attached psychological psychotherapists (*N* = 430; solid line) of our research from 2017/18 compared to the *N* = 1027 DACH 2017 reference sample (dotted line). All differences between the securely and insecurely psychotherapists are significant (*p* < 0.000) except the as “ns” designated ones. 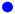 The bold dots designate the respective personality styles of the insecurely attached persons of the study of Sherry et al. (2007). 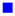 The bold quadrat corresponds with the result of Schauenburg et al. (2006) regarding depressive values of insecurely attached psychotherapists as well as those of the depressive FFI facets of the study of Both and Best (2017).

Single sample *t*-tests were used to determine whether the 80% (*n* = 342) securely attached psychotherapists differed from the 20% (*n* = 88) insecurely attached ones according to personality styles, which they actually did in 9 of the 14 personality styles. The results of the Levene- and *t*-tests and corresponding *p*-values and confidence intervals of the differences between these two groups can be found in Table 4. The mean T-scores of the personality styles of these two groups are depicted in Table 5. They as well as the means of the combined group of the *N* = 430 can also be seen as personality profiles in Figure 2 together with that of the reference group of the *N* = 1,027 DACH psychotherapists of 2015 (Peter et al., 2017). When comparing personality styles of securely attached (*n* = 342) to those of insecurely attached (*n* = 88) psychological psychotherapists, significant differences were found for the willful/paranoid (PN), spontaneous/borderline (BN), and reserved/schizoid (SZ) styles, as well as for the loyal/dependent (AB), self-critical/negativistic (NA), unselfish/self-sacrificing (SL), self-critical/avoidant (SU), passive/depressive (DE), and optimistic/rhapsodic (RH) styles. No significant differences were found in the ambitious/narcissistic (NA), the ominous/schizotypal (ST), assertive/antisocial (AS) charming/histrionic (HI), and conscientious/compulsive (ZW) styles (see Table 4 for details).

**TABLE 4:** Results of the Levene- and *t*-tests and corresponding confidence intervals of the differences between the two groups of the securely (*n* = 342) and insecurely (*n* = 88) attached psychotherapists.

**Levene test**								
**Personality styles**	**Variances are**	***F***	***p***	***T***	***df***	p* (two-tailed)	*Mean difference* ($\bar{x}{}_{1}$−$\bar{x}{}_{2}$)**	*SE of difference* ($\bar{x}{}_{1}$−$\bar{x}{}_{2}$)**
PN willful/**paranoid**	Equal	8.59	0.004	−5.92	428	0.000	−5.763	0.974
	Unequal			−5.16	116.567	0.000	−5.763	1.117
BL spontaneous/**borderline**	Equal	34.53	0.000	−6.81	428	0.000	−4.266	0.625
	Unequal			−5.29	105.881	0.000	−4.266	0.806
SZ reserved/**schizoid**	Equal	23.89	0.000	−4.14	428	0.000	−4.610	1.113
	Unequal			−3.37	109.932	0.001	−4.610	1.367
NA ambitious/**narcissistic**	Equal	3.84	0.051	−2.40	428	0.017	−2.175	0.905
	Unequal			−2.18	120.926	0.031	−2.175	0.999
AB loyal/**dependent**	Equal	9.14	0.003	−6.24	428	0.000	−5.741	0.920
	Unequal			−5.34	114.609	0.000	−5.741	1.074
NT critical/negativistic	Equal	4.10	0.043	−5.71	428	0.000	−4.728	0.828
	Unequal			−5.10	119.156	0.000	−4.728	0.927
ST intuitive/**schizotypal**	Equal	10.79	0.001	−1.59	428	0.113	−1.458	0.918
	Unequal			−1.37	115.371	0.173	−1.458	1.064
SL unselfish/self-sacrificing	Equal	7.04	0.008	−5.17	428	0.000	−4.997	0.967
	Unequal			−4.48	115.789	0.000	−4.997	1.116
SU self-critical/**avoidant**	Equal	2.06	0.152	−6.97	428	0.000	−6.106	0.876
	Unequal			−6.64	127.501	0.000	−6.106	0.920
DP passive/depressive	Equal	7.73	0.006	−8.39	428	0.000	−6.537	0.780
	Unequal			−7.17	114.434	0.000	−6.537	0.912
AS assertive/**antisocial**	Equal	4.92	0.027	−1.44	428	0.151	−1.357	0.942
	Unequal			−1.35	125.388	0.179	−1.357	1.005
HI charming/**histrionic**	Equal	5.43	0.020	2.24	428	0.026	2.368	1.057
	Unequal			1.96	116.998	0.052	2.368	1.207
RH optimistic/rhapsodic	Equal	0.47	0.494	4.35	428	0.000	4.268	0.982
	Unequal			4.08	125.662	0.000	4.268	1.045
ZW conscientious/**compulsive**	Equal	0.05	0.820	−0.75	428	0.452	−0.727	0.966
	Unequal			−0.75	133.938	0.456	−0.727	0.974

*^∗^ significant if *p* < 0.0018. ^∗∗^$\bar{x}{}_{1}$ = securely attached, $\bar{x}{}_{2}$ = insecurely attached. DSM-5 or ICD-10 equivalents are in bold print.*

**TABLE 5:** Data of the two groups of securely (*n* = 342) and insecurely (*n* = 88) attached psychotherapists.

**Personality styles**	**Attachment**	***n***	***Mean of T-values***	***SD***	***SE of mean***
PN willful/ **paranoid**	Secure	342	40.99	7.70	0.42
	Unsecure	88	46.75	9.72	1.04
BL spontaneous/ **borderline**	Secure	342	43.10	4.59	0.25
	Unsecure	88	47.36	7.19	0.77
SZ reserved/ **schizoid**	Secure	342	43.80	8.47	0.46
	Unsecure	88	48.41	12.08	1.29
NA ambitious/ **narcissistic**	Secure	342	43.22	7.28	0.39
	Unsecure	88	45.40	8.61	0.92
AB loyal/ **dependent**	Secure	342	43.84	7.20	0.39
	Unsecure	88	49.58	9.39	1.00
NT critical/negativistic	Secure	342	45.02	6.62	0.36
	Unsecure	88	49.75	8.03	0.86
ST intuitive/ **schizotypal**	Secure	342	45.99	7.21	0.39
	Unsecure	88	47.44	9.29	0.99
SL unselfish/self-sacrificing	Secure	342	45.82	7.61	0.41
	Unsecure	88	50.82	9.73	1.04
SU self-critical/ **avoidant**	Secure	342	46.55	7.20	0.39
	Unsecure	88	53.66	7.82	0.83
DP passive/depressive	Secure	342	46.26	6.09	0.33
	Unsecure	88	52.80	7.98	0.85
AS assertive/ **antisocial**	Secure	342	46.14	7.70	0.42
	Unsecure	88	47.50	8.58	0.91
HI charming/histrionic	Secure	342	47.55	8.37	0.45
	Unsecure	88	45.18	10.50	1.12
RH optimistic/rhapsodic	Secure	342	50.18	8.03	0.43
	Unsecure	88	45.91	8.92	0.95
ZW conscientious/ **compulsive**	Secure	342	48.68	8.06	0.44
	Unsecure	88	49.41	8.17	0.87

*DSM-5 or ICD-10 equivalents are in bold print.*

## Discussion

The data of our 2015 survey (Peter et al., 2017) indicated that the personality styles of *N* = 1,027 psychotherapists from the three German speaking countries Germany, Austria and Switzerland (DACH countries) differed from the norm (i.e., T-scores of 50 in Figure 1). In 2015 we had divided the personality styles into three groups according to the effect sizes of the differences. This resulted in reference data with which we can compare the data of the present sample of 2017/18. This time, in addition to the personality styles, we also asked for attachment styles from 430 psychological psychotherapist of the same DACH countries. We again used the PSDI by Kuhl and Kazén (2009) and added the RSQ by Griffin and Bartholomew (1994b).

Looking at Figure 2, it is obvious that the profile of the *n* = 342 securely attached psychotherapists of the present study (drawn through double line) – as compared with the insecurely attached (bold interrupted line) – is almost identical to the profile of the *N* = 1,027 DACH psychotherapists (dotted line) of 2015. As we had not considered attachment in 2015, an unknown number of insecurely attached psychotherapists was included in the whole sample. Judging by the minor deviations from the profile of the now *n* = 342 securely attached psychotherapists, these insecurely attached psychotherapists must not have been high in number. The *n* = 88 insecurely attached psychotherapists of the present sample also comprise only 20% of the total *N* = 430. This is similar to the rate of the German study by Taubner et al. (2014b) and that of the North American study by Fleischman and Shorey (2016), while dissimilar to the studies by the Heidelberg group (Dinger et al., 2009; Schauenburg et al., 2010).

The 20% insecurely attached psychological psychotherapists in the present sample (bold interrupted line) show a personality profile which differs significantly and adversely from that of the 80% securely attached in 9 of 14 styles. In the 2015 data, Peter et al. (2017) considered the first group of four personality styles (PN, BL, SZ, NA; see Figure 1) as *necessary* for building a good therapeutic relationship. In three of these four styles – willful/paranoid (PN), spontaneous/borderline (BL), and reserved/schizoid (SZ) – the insecurely attached individuals of the present sample scored significantly worse than their securely attached counterparts – even while they were still within the normal range between 40 and 60 and below the normative mean of 50 T-scores. Of the seven styles of the second group, which in 2015 we considered as *helpful* in the course of psychotherapy, the insecurely attached of the present sample likewise differed significantly in 5 of them, showing much higher, i.e., worse, values in the styles loyal/dependent (AB) and critical/negativistic (NT) – almost at the level of 50 –, and in unselfish/self-sacrificing (SL), self-critical/avoidant (SU), and passive/depressive (DE) – above the level of 50 T-scores. In the third group, which showed no differences to the norm in 2015, the insecurely attached participants of the present sample were significantly not as optimistic/rhapsodic (RH) as the securely attached, and, even though not significantly, they were not as charming/histrionic (HI) as their securely attached counterparts (see Figure 2).

It is intriguing that the results by Sherry et al. (2007) in insecurely attached persons are very similar to ours, as six personality styles of theirs match very well with five of ours, namely the styles paranoid, borderline, schizoid, dependent, and avoidant (cf the bold dots in Figure 2). The exception is schizotypal because we didn’t find any difference regarding this style; another exception are Sherry et al.’s values in the histrionic style for the insecurely attached which is similar to ours. However, they did not reach the required level of significance (Table 4 and Figure 2). Sherry et al.’s (2007) personality data was collected using Millon’s Inventory and is therefore not directly comparable to our data surveyed using the PSDI, although a high correspondence of both results is apparent. This is also true for the results by Both and Best (2017) who, using the NEO-PI-R, found low neuroticism and high extraversion for the securely attached, versus high neuroticism, low extraversion, and low agreeableness for the insecurely attached, as well as high depression and low trust (see Table 3). This corresponds very well with our PSDI values of the paranoid (PN), borderline (BL), schizoid (SZ), dependent (AB), negativistic (NT), avoidant (Su), and depressive (DP) personality styles, as Kuhl and Kazén (2009) found meaningfully significant correlations between these styles and the factors neuroticism, extraversion, and agreeableness of the NEO-FFI (see Table 6, which we placed closely above Figure 2, in order to make the correspondence visible). Additionally, the results by Schauenburg et al. (2006) correspond with ours, as our insecurely attached individuals differed most from the securely attached in the passive/depressive (DP) style (see the bold quadrat in Figure 2). Moreover, it is of note that the results by Sherry et al. (2007) as well as those by Both and Best were drawn from young undergraduates, while the results by Schauenburg et al. (2006) as well as our own refer to older professional psychotherapists, meaning that the correlation of personality with attachment styles is probably a stable one.

**TABLE 6:** Correlations between NEO-FFI and PSDI values (Kuhl and Kazén, 2009).

**NEO\PSDI**	**PN**	**BL**	**SZ**	**NA**	**AB**	**NT**	**ST**	**SL**	**SU**	**DP**	**AS**	**HI**	**RH**	**ZW**
Neurotic	0.27	0.66	0.20		0.44	0.27			0.67	0.73				
Extraver	−0.30	−0.28	−0.57			−0.30	0.21		−0.28	−0.36		0.25		
Agreeab	−0.31		−0.44	−0.23		−0.21								0.34
Conscien		−0.26				−0.41				−0.29				
Openess							0.29							

## Conclusion

Successful outcomes in psychotherapy depend on many factors such as technical interventions, patients’ pathology and characteristics, and therapeutic relationship or alliance, respectively. The therapeutic relationship has been the topic of extensive theoretical and practical elaborations since the beginnings of psychotherapy in 1775 (Peter and Revenstorf, 2018) and have received more attention in psychotherapy research at least since the end of the last century (Horvath and Symonds, 1991). Therapeutic relationship and alliance are highly dependent on the psychotherapist. As early as 1986, Luborsky et al. (1986) stated, that “variations in success rate typically have more to do with the therapist than with the type of treatment.” This statement on the therapist variable has been confirmed by others (Baldwin et al., 2007; Dinger et al., 2008; Zuroff et al., 2010; Norcross and Lambert, 2018; Norcross and Wampold, 2018). Parts of the therapist variable are concerned with personality and attachment styles of therapists. Therapist’s attachment status is obviously highly associated with his/her ability to attune to patients and this influences the therapeutic process considerably (Talia et al., 2018). Also, according to Lingiardi et al. (2017), there is primary evidence that therapists’ attachment styles, their interpersonal history with caregivers, and their self-concept influences treatment outcome. Peter et al. (2017) published the first study which correlated therapists’ personality styles with a public norm and suggested that special personality styles of the therapists’ may be *necessary* and others *helpful* for forming a good therapeutic alliance. In the present study we correlated personality and attachment styles and confirmed the results by Both and Best (2017), Schauenburg et al. (2006), and Sherry et al. (2007) who also found significant differences between securely and insecurely attached psychotherapists. From this, one could infer that personality and attachment styles are crucial factors for being or becoming a good psychotherapist if one considers them to be strong moderators for therapeutic alliance with the latter being a strong factor for therapeutic outcome (Baldwin et al., 2007). However, this would be a kind of assertion which has not yet been proven. Such an assertion might be overly generalized as it is not substantiated by our study. So we must be careful to attribute attachment such a decisive role for psychotherapeutic practice. As long as we lack data attachment can be only considered a moderator variable.

## Limitations and Implications

There are many limitations of our study which we have already discussed extensively in Peter et al. (2017). What is more, because all our data was collected from German speaking psychotherapists of the DACH countries a cultural bias must be presumed. The transfer of applying the results and implications to other sociocultural contexts is therefore problematic. First of all, however, we had no chance in our anonymous online survey to receive data about outcome. This we consider a main limitation to the implications of our study. Yet, also Sherry et al. (2007) as well as Both and Best by examining young undergraduates (of 22,15 and 20,54 years in the mean) did not touch upon this outcome issue, and even Schauenburg et al., 2006 who used psychotherapists (mean 37,4 years) did not report on outcome. In a later study, however, Schauenburg et al. (2010) found no general main effects of therapists’ attachment styles to predict alliance and outcome with the exception of more severely impaired patients, where higher attachment security of the therapist was associated with both better alliance and outcome. So, we should be careful regarding our study’s implications on the personal and professional quality of our psychotherapists.

It may be that there are some personality styles that predispose for wishing to become a psychotherapist (Prade et al., 2014) and then to be a good psychotherapist. Others may be unfavorable. Insecure attachment styles obviously correlate with personality styles which are – according to the results of Peter et al. (2017) – not favorable to form a good therapeutic relationship. Yet, are they indicative of being a bad psychotherapist? Many educational factors in the course of the training and then being a psychotherapist may compensate for possible adverse personality and attachment styles (Rizq and Target, 2010). The extensive self-analysis of the psychoanalysts and the profound self-explorations of psychodynamic psychotherapists are examples of these kinds of compensatory measures. The relevance of the alliance factor is acknowledged in almost all of today’s psychotherapy approaches (Fiedler, 2018). Large parts of the education in client-centered therapy and Ericksonian hypnotherapy for example were and are devoted to verbal and non-verbal skills to get in contact with a patient, to establish a good therapeutic relationship and to use this “rapport” for the benefit of the patients (Revenstorf and Peter, 2015). Personal therapy is used by many psychotherapists of all orientations, and supervision and continuous education in the course of psychotherapeutic practice is a matter-of-course requirement (Bike et al., 2009; Orlinsky et al., 2011). Just one pointer toward this educational hypothesis would be our sample’s mean age of 57.1 years, meaning that the 20% of the insecurely attached have had enough time and options to readapt their unfavorable styles to the needs of their profession. So, training, continuous education, self-exploration, experience, and supervision may compensate for therapists’ attachment deficits and may serve as an acquired resource for therapeutic effectiveness. It has been shown that attachment representations can be improved by psychotherapy (Taylor et al., 2015; Buchheim et al., 2017). Therefore, future studies might be able to demonstrate whether it is possible, in the process of psychotherapy education, to change an insecure attachment style essentially so that the respective personality styles match with that of securely attached psychotherapists. This, then probably, would result in a kind of “earned security” (Pearson et al., 1994). If not, would it be possible to show that, at least, the *“rapport” behavior* could be changed in order to enable psychotherapists to establish and maintain a good therapeutic alliance. Applying PSDI and RSQ measurements at the beginning and the end of psychotherapy education curricula could help to answer those questions and to provide replication data for our study.

## Data Availability Statement

The raw data supporting the conclusions of this article will be made available by the authors, without undue reservation, to any qualified researcher.

## Ethics Statement

In accordance with the Declaration of Helsinki and local legislation, no formal institutional approval was sought because of the very nature of the study (online survey targeting adults and using a transparent, non-offending personality questionnaire): Psychotherapists were contacted by e-mail addresses and informed openly about the aim and purpose of the study. They filled in the questionnaire anonymously and received no compensation for participation. Therefore, no additional written consent was sought other than the consent expressed by participating.

## Author Contributions

BP conceptualized the study, organized the data collection and study setup, and wrote the manuscript. EB performed the statistical analysis and contributed substantially to writing the method and results section.

## Conflict of Interest

The authors declare that the research was conducted in the absence of any commercial or financial relationships that could be construed as a potential conflict of interest.
